# The central inflammasome adaptor protein ASC activates the inflammasome after transition from a soluble to an insoluble state

**DOI:** 10.1016/j.jbc.2022.102024

**Published:** 2022-05-11

**Authors:** Evan R. Prather, Mikhail A. Gavrilin, Mark D. Wewers

**Affiliations:** Division of Pulmonary Critical Care and Sleep Medicine, Davis Heart and Lung Research Institute, Ohio State University Wexner Medical Center, Columbus, Ohio, USA

**Keywords:** inflammasome, ASC, oligomerization, caspase-1, IL-1β, afc, 7-amino-4-trifluoromethylcoumarin, ASC, apoptosis-associated speck-like protein containing a CARD, CARD, caspase recruitment domain, IL-1β, interleukin-1β, IVAS, *in vitro* model of inflammasome activation, KI, knocked-in, LDH, lactate dehydrogenase, LPS, lipopolysaccharide, MBP, maltose-binding protein, P, insoluble, PYD, pyrin domain, rASC, recombinant ASC, S, soluble, TBST, Tris-buffered saline with Tween-20, WEHD, Trp-Glu-His-Asp

## Abstract

Apoptosis-associated speck-like protein containing a caspase recruitment domain (CARD) (ASC) is a 22 kDa protein that functions as the central adaptor for inflammasome assembly. ASC forms insoluble specks in monocytes undergoing pyroptosis, and the polymerization of ASC provides a template of CARDs that leads to proximity-mediated autoactivation of caspase-1 in canonical inflammasomes. However, specks are insoluble protein complexes, and solubility is typically important for protein function. Therefore, we sought to define whether ASC specks comprise active inflammasome complexes or are simply the end stage of exhausted ASC polymers. Using a THP-1 cell–lysing model of caspase-1 activation that is ASC dependent, we compared caspase-1 activation induced by preassembled insoluble ASC specks and soluble monomeric forms of ASC. Unexpectedly, after controlling for the concentration dependence of ASC oligomerization, we found that only insoluble forms of ASC promoted caspase-1 autocatalysis. This link to insolubility was recapitulated with recombinant ASC. We show that purified recombinant ASC spontaneously precipitated and was functional, whereas the maltose-binding protein–ASC fusion to ASC (promoting enhanced solubility) was inactive until induced to insolubility by binding to amylose beads. This functional link to insolubility also held true for the Y146A mutation of the CARD of ASC, which avoids insolubility and caspase-1 activation. Thus, we conclude that the role of ASC insolubility in inflammasome function is inextricably linked to its pyrin domain–mediated and CARD-mediated polymerizations. These findings will support future studies into the molecular mechanisms controlling ASC solubility.

Apoptosis-associated speck-like protein containing a caspase recruitment domain (CARD) (ASC) was first discovered using monoclonal antibodies generated against proteins in the insoluble fraction of apoptotic promyelocytic leukemia cells (1). One specific antibody facilitated the cloning of a CARD and pyrin domain (PYD) containing 22 kD protein that formed speck-like aggregates in apoptotic HL60 cells, named ASC. Subsequently, ASC was found to be integral to the inflammasome, providing the central adaptor for the entire protein complex. ASC oligomers induce caspase-1 dimerization and proximity-induced activation (2, 3, 4).

Since the original description of the inflammasome (5), many inflammasome complexes have been discovered with the majority being ASC dependent (6). In ASC-dependent inflammasomes, ASC is recruited to a pattern-recognition protein, typically of the nucleotide oligomerization domain–like receptor family (7). Early during this recruitment process, ASC oligomerizes and displays CARD filaments that recruit caspase-1 CARDs into proximity-mediated activation while forming the ASC speck. However, the relationship between the insoluble ASC speck and the functional inflammasome construct is incompletely known (4, 8). While it is known that the ASC speck is a marker of inflammasome activation (9, 10), there are conflicting models to describe at what stage of ASC oligomerization caspase-1 binds and autocleavage occurs (11, 12, 13, 14, 15, 16, 17, 18). We hypothesized that the optimum ASC timing for caspase-1 activation would be early in the oligomerization stage when soluble PYD–PYD ASC filaments form, displaying arrays of available uncomplexed CARDs (4). Such formations would promote caspase-1–CARD binding to the ASC–CARD and thus induce a proximity-mediated autoactivation of caspase-1. As a corollary, we reasoned that the end-stage, consolidated, insoluble, and eccentrically placed ASC speck might no longer display available CARDs because of ASC–ASC CARD interactions that sterically hinder caspase-1 recruitment and thus impede proximity-mediated caspase-1 dimerization. Thus, expanding on this, we set out to study the relationship between soluble and insoluble ASC in the activation of caspase-1 by using a cell-free model (19) that allowed the study of ASC in its various states.

## Results

### *In vitro* assembled ASC specks activate caspase-1 and lead to interleukin-1β processing

**Figure 1:**
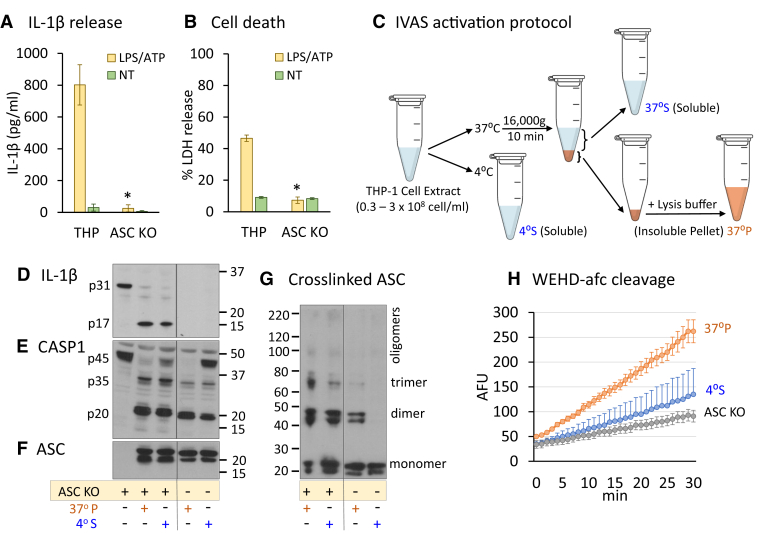
**Late-stage ASC specks maintain capacity for activation of caspase-1.** ASC-dependent inflammasome activation was confirmed in THP-1 WT and THP-1 ASC KO cells not treated (NT) or treated with 1 μg/ml LPS (3–4 h) and then 5 mM ATP (30 min) by IL-1β ELISA (n = 7 independent experiments) (*A*) and LDH assay (n = 3 independent experiments) (*B*), ∗*p* < 0.05 as ASC KO compared with THP-1 WT cells. Cell-free *in vitro* ASC specks (IVAS) were created using high cell lysate concentrations at 37 °C, and the soluble and insoluble fractions were separated by centrifugation at 16,000*g* for 10 min (*C*). These 4 °S and 37 °P fractions were then separately combined 1:1 with LPS-primed THP-1 ASC KO lysate, warmed at 37 °C for 30 min, whereas the samples not added to ASC KO lysates were *left* on ice. Resultant fractions were then immunoblotted for IL-1β cleavage (*D*), caspase-1 cleavage (*E*), total ASC (22 and 20 kDa splice variants) (*F*), and DSS cross-linked ASC before and after addition to ASC KO lysate (*G*) (*D*–*G* independently repeated seven times). Separately, the fractions were assayed for caspase-1 activity at room temperature by WEHD-afc cleavage (*H*) (n = 3 independent experiments for THP-1 and n = 6 independent experiments for ASC KO). Data are presented as mean fluorescent units (AFUs) ± SEM. ASC, apoptosis-associated speck-like protein containing a CARD; DSS, dextran sodium sulphate; IL-1β, interleukin 1β; LDH, lactate dehydrogenase; LPS, lipopolysaccharide; WEHD-afc, Trp-Glu-His-Asp-7-amino-4-trifluoromethylcoumarin.

To study the relative function of ASC oligomers in their soluble and insoluble forms, we utilized WT and an ASC KO strain of THP-1 cells in an established *in vitro* model of inflammasome activation (termed IVAS) (20). First, we confirmed that our cells responded to a nucleotide oligomerization domain–like receptor P3-specific trigger in an ASC-dependent fashion (Fig. 1, *A* and *B*). Next, we lysed WT THP-1 cells at 3 × 10^8^ cells/ml and used this cell extract to create soluble (S) and insoluble (P) ASC complexes as previously described (19, 21) (Fig. 1*C*). These extracts were then incubated with the lysate from primed ASC KO cells (“ASC KO substrate”), which provided pro–interleukin-1β (IL-1β) and further procaspase-1 substrate. We confirmed that ASC is oligomeric in the warmed insoluble fraction (37 °P) and monomeric in the cold soluble fraction (4 °S) prior to incubation with the ASC KO substrate (Fig. 1*G*). Furthermore, the total amount of ASC in these two fractions was similar (Fig. 1, *F* and *G*).

After addition to the ASC KO substrate, both samples induced cleaved caspase-1 and IL-1β. Although the 4 °S sample did induce activation of procaspase-1 as expected, the 37 °P addition showed enhanced activity in comparison (Fig. 1, *D* and *E*). Unfortunately, the 4 °S ASC oligomerized when added to the warmed ASC KO substrate (Fig. 1*G*), making it impossible to distinguish its monomer from its oligomer function. (Note that proIL-1β was only provided by the primed ASC KO substrate.) Furthermore, when the 37 °P and 4 °S fractions were tested on a synthetic substrate (*i.e.*, WEHD [Trp-Glu-His-Asp]-afc [7-amino-4-trifluoromethylcoumarin] [Calbiochem], a substrate specific for caspase-1/4/5 (22), the 37 °P fraction consistently generated more substrate cleavage than the 4 °S fraction [Fig. 1*H*]). Thus, insoluble ASC specks productively generate an active inflammasome from ASC KO lysates and therefore are not simply end-stage products of completed inflammasome activation. We therefore turned our attention to the solubility status of ASC as it relates to caspase-1 function.

### *In vitro* caspase-1 processing is dependent on ASC oligomerization status

**Figure 2:**
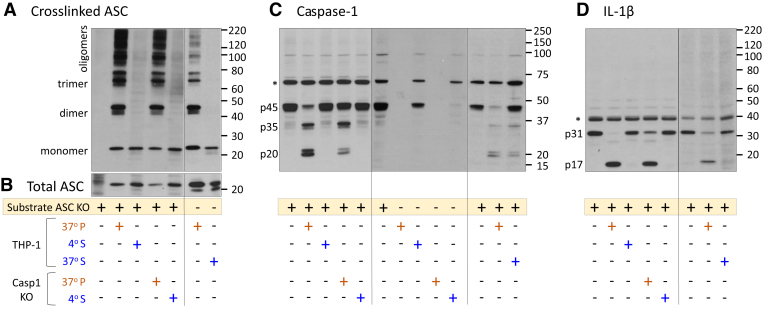
**Active ASC is insoluble and oligomeric.** WT and Casp1 KO THP-1 cells were lysed at 1 × 10^8^ cells per ml, and their corresponding 37 °P, 4 °S, and 37 °S fractions were generated as noted for Figure 1*C*. These fractions from both, WT and Casp1 KO THP-1, cells were incubated 1:1 with lysates from LPS (1 μg/ml) primed ASC KO cells and analyzed for oligomers of ASC (*A*), total ASC (*B*), cleavage of caspase-1 (*C*), and processing of proIL-1β (*D*). Immunoblots are representative of three independent experiments. *Asterisk* in *C* and *D* shows nonspecific binds. ASC, apoptosis-associated speck-like protein containing a CARD; IL-1β, interleukin 1β; LPS, lipopolysaccharide.

Because ASC in the 4 °S fraction oligomerized when added to warm ASC KO cell lysates (Fig. 1*G*), it was not possible to distinguish soluble from insoluble ASC roles for this activity. To better control the caspase-1 activation potential of soluble *versus* insoluble ASC, we made several modifications to our IVAS activation protocol (Fig. 1*C*). First, in order to maintain ASC in a soluble form during inflammasome activation, we lowered the cell concentrations in the IVAS activation protocol (Fig. 1*C*) to 1 × 10^8^ cells/ml, the threshold concentration for IVAS-mediated caspase-1 activation (20). Lysing below 1 × 10^8^ cells/ml failed to generate detectable ASC oligomers in immunoblots (Supporting Information). Second, in order to isolate the caspase-1 activation to the caspase-1 from the ASC KO substrate, THP-1 caspase-1 KO lysate fractions were used to generate ASC specks. Third, we included the 37 °S fraction in selected samples (*i.e.*, the supernatant of the 37 °P fraction containing soluble unpelleted ASC). As expected, ASC immunoblots confirmed that ASC, in both the 4 °S and 37 °S fractions, remained monomeric after addition to ASC KO substrate. Whereas in contrast, the 37 °P samples from both cell types (WT and caspase-1 KO) generated oligomerized ASC (Fig. 2*A*). Importantly, total ASC was similar between the 37 °P and 4 °S fractions (Fig. 2*B*). The caspase-1 immunoblot (Fig. 2*C*) showed depleted p45 procaspase-1 and detectable p35 and p20 cleaved caspase-1 bands in the 37 °P additions from both THP WT and caspase-1 KO fractions. In comparison, only p45 procaspase-1 was present in the 4 °S additions. The cleaved caspase-1 in the 37 °P additions was exclusively from ASC KO substrate, as caspase-1 was not detected in either 37 °P fraction. Notably, while the overall intensity of cleaved caspase-1 signal in Figure 2*C* (third blot) was reduced, complete cleavage of p45 only occurred with the 37 °P addition. Correspondingly, IL-1β was also only cleaved by the 37 °P addition but not by the 4 °S or 37 °S additions, for both WT and caspase-1 KO fractions (Fig. 2*D*). Hence, when soluble and insoluble forms of ASC were compared head to head, only insoluble forms of ASC proved to be capable of activating caspase-1.

### Procaspase-1 processing depends upon the oligomerization of ASC *via* PYD

Although ASC was originally discovered for its tendency to insolubility (1), relatively little attention has been placed on how the solubility of ASC relates to caspase-1 activation. In this context, it is important to note that both PYD and CARDs of ASC are independently capable of forming filaments when expressed individually (23). We therefore compared caspase-1 activation in our cell-free model using ASC constructs with either PYD or CARD modifications.

**Figure 3:**
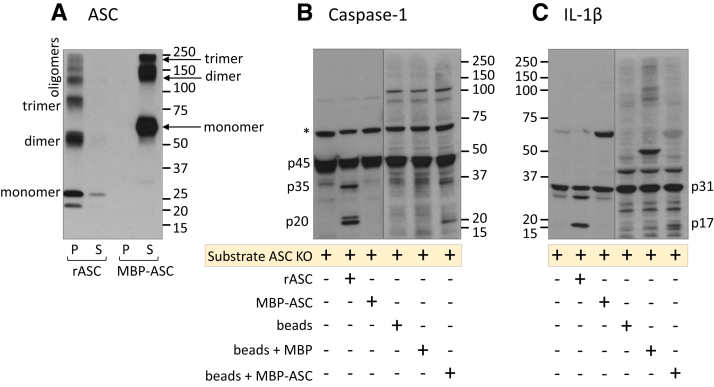
**Solubility status of recombinant ASC (rASC) determines its functional capability.** rASC or MBP-ASC (350 ng at 120 μg/ml) was warmed at 37 °C for 30 min, and the insoluble (P) and soluble (S) fractions, prepared as described for IVAS. Both were tested in the *in vitro* activation assay, and then, fractions were separated and treated with DSS before immunoblotting for ASC (*A*). To generate primed ASC-free substrate, ASC KO cells were primed with 1 μg/ml of LPS to induce proIL-1β and then incubated alone or with rASC, MBP-ASC, amylose beads alone, amylose beads with MBP alone, or amylose beads with MBP-ASC (350 ng ASC in rASC, MBP-ASC and bead + MBP-ASC fractions) at 37 °C for 30 min and then immunoblotted for caspase-1 cleavage (*B*) and IL-1β cleavage (*C*) (*A*–*C* representative of duplicate experiments). *Asterisk* represents nonspecific band. ASC, apoptosis-associated speck-like protein containing a CARD; DSS, dextran sodium sulphate; IL-1β, interleukin 1β; IVAS, *in vitro* model of inflammasome activation; MBP, maltose-binding protein.

We first studied recombinant ASC (rASC) alone or rASC with a maltose-binding protein (MBP) tag at the aminoterminal PYD of ASC. We noted that stock solutions of rASC (Origene) tended to precipitate spontaneously, whereas MBP-ASC remained soluble. Since an aminoterminal MBP tag inhibits ASC PYD peptides from spontaneous filament formation (24), MBP-ASC represents a unique form of ASC that prevents PYD filament formation and remains soluble. While our gel format cannot detect large MBP-ASC oligomers, we did observe MBP-ASC dimers and trimers that did not precipitate with warming (Fig. 3*A*), whereas THP-1-derived ASC and rASC oligomers were found exclusively in the pelleted insoluble fraction. Consistent with previous results, the pelleted rASC activated ASC KO procaspase-1 to cleave proIL-1β, but dimers and trimers of soluble MBP-ASC did not (Fig. 3, *B* and *C*). These data suggest that ASC activity may be characterized not only by ASC oligomerization but also by its solubility state.

To further support the solubility status of ASC as a critical factor in caspase-1 processing, we rescued the activity of MBP-ASC by immobilizing MBP-ASC to amylose beads as a form of insolubility that corrects for any disrupted PYD oligomerization caused by the aminoterminal MBP. While amylose beads alone or amylose beads bound to MBP alone produced no cleaved caspase-1 or IL-1β, amylose beads bound to MBP-ASC yielded cleaved forms of caspase-1 and IL-1β (Fig. 3, *B* and *C*). This result is consistent with the hypothesis that oligomerization of ASC *via* its PYD is critical for its caspase-1 activation function and its tendency for precipitation.

### Y146A mutation of ASC CARD inhibits transition from soluble to insoluble state, thereby disrupting caspase-1 cleavage

Because ASC speck formation is dependent upon both its PYD and CARD interactions (23, 25), we next turned to the carboxyterminal CARD of ASC. We used a modified ASC CARD construct to investigate the CARD role of ASC in ASC solubility and caspase-1 function. We recently reported that the CARD mutant Y146A-ASC is deficient in speck formation and caspase-1 activation (26). Although Y146A-ASC does form fine filamentous structures after transfection (likely *via* PYD filamentation), it does not form insoluble specks. This lack of precipitation is consistent with an inability of Y146A-ASC’s CARD to bind to its CARD dimer pair, thus failing to complete the consolidation of ASC with its primary PYD/PYD filament, an event required for speck formation (23, 25).

**Figure 4:**
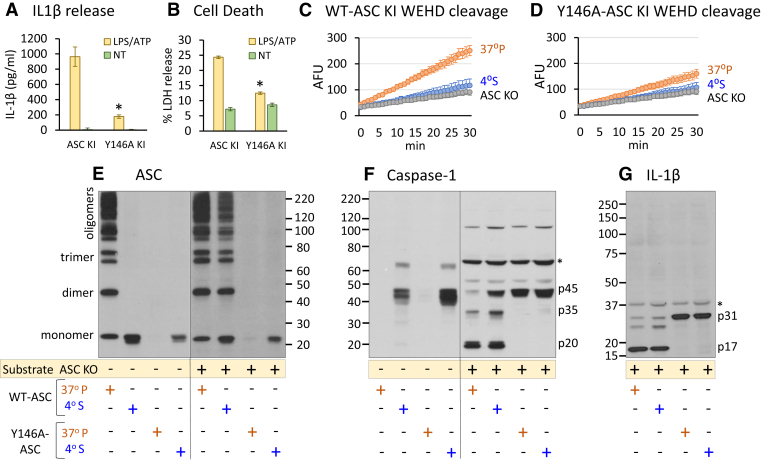
**ASC tyrosine 146 linked to ASC solubility and caspase-1 activation.** THP-1 ASC KO cells were knocked-in with either WT ASC or Y146A ASC to confirm pyroptotic dysfunction in the Y146A KI cells as assessed by IL-1β release (sandwich ELISA) (n = 8 independent experiments) (*A*) and LDH release (n = 3 independent experiments) (*B*), ∗*p* < 0.05 as Y146A KI compared with ASC KI. Caspase-1 activity was measured by WEHD-afc cleavage between the 4oS fraction and the 37 °P fraction (n = 3 independent experiments) (*C* and *D*). Data are expressed as mean fluorescent units (AFUs) ± SEM. The soluble and insoluble fractions of the WT and Y146A KI cells were incubated alone or added to primed ASC KO lysate 1:1, warmed at 37 °C for 30 min, and then immunoblotted for cross-linked ASC (*E*), cleaved caspase-1 (*F*), and IL-1β cleavage (after addition to ASC KO only) (*G*) (*E*–*G* n = 3 independent experiments). *Asterisks* represent nonspecific bands. ASC, apoptosis-associated speck-like protein containing a CARD; IL-1β, interleukin 1β; KI, knock-in; LDH, lactate dehydrogenase; WEHD-afc, Trp-Glu-His-Asp-7-amino-4-trifluoromethylcoumarin.

Considering the role of the solubility of ASC, we hypothesized that filamentous mutant ASC remains soluble after activation, whereas tightly packed and consolidated WT-ASC becomes insoluble. To test this idea, we knocked-in (KI) WT and Y146A ASC into our ASC KO cells. Using these KI cells, we confirmed that WT ASC KI restores inflammasome function, whereas the mutant Y146A-ASC disrupts IL-1β and lactate dehydrogenase (LDH; Roche Applied Science) release in response to lipopolysaccharide (LPS; InvivoGen) + ATP treatment (Fig. 4, *A* and *B*). We performed our cell-free activation protocol at our original lysing concentration (3 × 10^8^ cells/ml) to provide a suitable concentration of caspase-1 to measure WEHD-afc kinetics as we described in Figure 1*H*. We observed that WEHD-afc cleavage was increased in the WT ASC KI 37 °P fraction compared with the 4 °S fraction and the ASC KO control (Fig. 4*C*), as we had similarly observed in THP-1 lysates (Fig. 1*C*). This difference was diminished in the Y146A-ASC 37 °P fraction compared with the Y146A-ASC 4 °S fraction (Fig. 4*D*). An immunoblot of these samples found WT-ASC KI oligomers present in the 37 °P fraction, whereas only monomeric ASC was detected in the 4 °S fraction. As expected, no ASC was detected in the insoluble 37 °P fraction from the Y146A-ASC KI cell extract, whereas only monomeric Y146A-ASC was detected in the 4 °S fraction. Upon addition to ASC KO cell extract, WT-ASC KI 4 °S formed oligomers but Y146A-ASC KI 4 °S did not (Fig. 4*E*).

Accordingly, when added to ASC KO lysate 1:1, WT-ASC KI 37 °P depleted all procaspase-1, and the WT-ASC KI 4 °S was slightly less effective (Fig. 4*F*). On the other hand, neither the 37 °P nor the 4 °S Y146A-ASC fractions depleted procaspase-1 or generated p35 or p20 forms following ASC KO lysate addition. In all cases, caspase-1 cleavage events corresponded with IL-1β maturation (Fig. 4*G*). Thus, the CARD dysfunction of Y146A-ASC correlated with its predisposition to solubility, consistent with the knowledge that ASC CARD–CARD interactions are critical to completing speck formation (23, 25).

## Discussion

Our studies utilized a classic cell-free system that allows the study of caspase-1 activation in a controlled environment (5, 19), while taking advantage of ASC KO and KI models. We show that lysing THP-1 cells in a hypotonic buffer produces activation of caspase-1 in an ASC-dependent manner that was unexpectedly closely linked to its insolubility.

The project initially set out to test the hypothesis that ASC, the central adaptor for most inflammasome structures, provides protein/protein interfaces that lead to caspase-1/5 activation well before ASC has collapsed to its classic insoluble speck form. We had hypothesized that ASC specks represent the end stage of inflammasome activity and not the functional activation phase. We reasoned that although ASC was discovered because of its insolubility in apoptotic cells (1), most proteins are not functional when insoluble. For example, protein insolubility presents a critical problem in the pharmaceutical development of functional recombinant proteins (27). Second, ASC rapidly collapses into a single paranuclear speck that could geometrically and physically limit access of caspase-1 to ASC oligomers (28). Last, host insoluble or misfolded proteins are typically linked to disease states, not physiological functions. For example, prion-driven dementias, amyloidosis, and genetically induced protein folding disorders (*e.g.*, alpha 1-antitrypsin deficiency (29) and cystic fibrosis (30)) are all pathological events caused by insoluble or misfolded proteins. Nevertheless, our experiments failed to support this conceptual framework but instead demonstrated robust activation capacity of ASC in its insoluble speck configuration.

The evidence for the function of the ASC speck was first shown by comparing the ability of soluble and insoluble forms of ASC to promote caspase-1 autoactivation and proIL-1β cleavage when combined with THP-1 ASC KO cell lysates (Fig. 1). Both ASC forms showed this capacity. Insoluble specks induced caspase-1 function but so did the ASC in the 4 °S (supernatant of the cell extract). Therefore, we needed to determine if the 4 °S sample actually remained soluble when mixed with the ASC KO cell lysate. But as shown in Figure 1*G*, the highly concentrated THP-1 cold fraction (4 °S) of ASC oligomerized when added to ASC KO cell lysates and warmed.

To control for this solubility issue, we diluted the cell concentration to the threshold for ASC oligomerization, so that addition of the soluble ASC to ASC KO lysate would dilute it below this threshold, and ASC would not spontaneously oligomerize (Supporting Information). We then compared ASC oligomers to ASC soluble fractions at this concentration (Fig. 2). In this setting, only the insoluble oligomeric forms of ASC, free of caspase-1 carryover, were able to activate caspase-1 and cleave proIL-1β. Thus, insolubility relates directly to the function of ASC.

To further test the solubility hypothesis, we turned to rASC in place of the THP-1 cell lysate ASC source. Consistent with the requirement for ASC insolubility, *Escherichia coli* generated rASC-formed oligomers and was perfectly capable of activating caspase-1 when added to ASC KO THP-1 cell lysates. On the other hand, ASC, with its PYD linked to MBP (to promote ASC solubility), was unable to activate caspase-1 despite being capable of forming trimers. However, when induced to insolubility by linking MBP-ASC to amylose beads, MBP-ASC reacquired caspase-1 activation (Fig. 3).

Last, we recently noted that tyrosine 146 (CARD) of ASC partially regulates the function of ASC (26), which agrees with structural studies that link the CARD of ASC to caspase-1 CARD filaments (31). Thus, we elected to determine if this effect might relate to ASC solubility (Fig. 4). As we show, the ASC Y146A mutant largely remains soluble when subjected to our *in vitro* activation protocol, and this predisposition to solubility correlates with the need for both PYD and CARD functional domains and further supports the concept that ASC functions only in its highly concentrated insoluble state. Hence, ASC insolubility requires both a functional PYD and a functional CARD. Their combined effects on speck formation (insolubility) appear to be critically linked to the function of ASC in inflammasome activation.

There are however limitations of this study that require comment. Our activation format for caspase-1 relies on a cell-free system that may not fully recapitulate the *in vivo* activation functions of ASC. For example, studies have noted minimal to no ASC speck formation in some settings with human monocytes actively secreting IL-1β, suggesting alternative paths for inflammasome activation (13, 14, 18, 32). Second, we cannot exclude the possibility that the dysfunction of the soluble MBP-ASC effect on caspase-1 is due to impeded PYD filament formation. However, it is just as likely that the inability to form the primary PYD filament (a requirement for speck formation) is also the functional link to insolubility. Furthermore, our MBP-ASC model of soluble polymerization could not confirm oligomers larger than trimers because of limitations in our gel format for complexes larger than 220 kDa. In addition, it has yet to be documented that trimers of ASC in any form provide sufficient template for caspase-1 autoactivation. Finally, we recognize that the boundary between solubility and insolubility is difficult to define. It is conceivable that the growing edge of the enlarging ASC speck is the active site for caspase-1 proximity-based activation as has been suggested (4) and that the water-facing edge of the ASC speck provides the template for caspase-1 recruitment and dimerization as noted previously (23). Thus, one could argue that ASC specks represent a dichotomous complex with both insoluble and soluble aspects.

In hindsight, there were additional signs that our original bias against insolubility would not be supported by experimentation. For one, it is clear that ASC has an enormous stoichiometric advantage over caspase-1 (we estimate 135 mol of ASC per mole of caspase-1 based upon published measures of caspase-1 and ASC per cell (19, 33)). Hence, it might be difficult to quench all CARD/CARD interactions within the ASC speck. In addition, electron microscopic studies of the filament bundles of ASC (23) reveal lateral branches off the ASC lattice formation that provide freshly available CARDs to capture procaspase-1 and induce proximity-mediated activation.

In summary, contrary to our original working hypothesis, the tendency of ASC to insolubility appears to be inextricably linked to its function to promote inflammasome activation. Future studies dissecting the molecular details that regulate this ASC transition state may offer novel therapeutic angles to prevent inflammasome-mediated pathologies.

## Experimental procedures

### Activation experiments

THP-1, ASC KO, WT-ASC KI, or Y146A-ASC KI modified cells, as previously reported (18, 26), were incubated in RPMI1640 media + GlutaMax (Life Technologies) containing 10% heat-inactivated fetal bovine serum (Atlas Biologicals) and 1% penicillin/streptomycin (Gibco) at 5 × 10^6^ cells/ml. In select experiments as denoted, the inflammasome substrate for our activation experiments, ASC KO cells, was primed with 1 μg/ml LPS for 3 h to trigger transcription of proIL-1β (34). This model allows inflammasome proteins to be primed and preactivated without final assembly *via* ASC adaptor into active caspase-1 and IL-1β. Cells were then collected and washed twice in PBS (Gibco) before lysing in buffer W (20 mM Hepes [pH 7.48], 10 mM KCl, 1.5 mM MgCl_2_, 1 mM EGTA, and 1 mM EDTA) supplemented with a protease inhibitor cocktail (Millipore; P8340-5ML), PMSF, 100 μM neutrophil elastase inhibitor methoxy-succinyl-ala-ala-pro-val-chloromethyl ketone (Sigma–Aldrich), and 1,4-DTT. Each cell type was combined to 30 × 10^6^ cells (Fig. 1, *D*–*H*) or 10 × 10^6^ cells (Fig. 2) and lysed on ice in 100 μl of buffer W using three sets of 10 strokes from a 22-gauge needle with a tuberculin syringe (intervals done to avoid heating). Mechanical lysis was done without the presence of a strong detergent so that *in vitro* activation of caspase-1 and IL-1β would not be inhibited. Cell debris was removed by centrifugation at 16,000*g* for 10 min at 4 °C; cell extract was separated from the insoluble cell pellet and transferred to a prechilled tube to avoid spontaneous ASC oligomerization. Half of this cell extract was warmed to 37 °C for 30 min to promote *in vitro* assembly of ASC specks, and the other half was kept on ice as a speck-free control (4 °S), as we described in detail earlier (21). Following incubation, the warmed cell extract was spun down at 16,000*g* for 10 min, and the supernatant fraction (37 °S) was removed from the insoluble pellet (37 °P), which was resuspended in lysing buffer to the original volume (the protocol is outlined in Fig. 1*C*). THP-1, ASC KI, or Y146A KI insoluble or soluble ASC was combined 1:1 with ASC KO lysate (inflammasome activation substrate) and incubated at 37 °C for 30 min. The control 4 °S and 37 °P samples, that is, those not added to the ASC KO lysates, were kept on ice during this 30 min incubation.

For the rASC experiments, 350 ng MBP-ASC (purified in our laboratory) or rASC (Origene) was combined with ASC KO lysate and incubated at 37 °C for 30 min. Amylose beads (New England Biolabs) were washed five times in buffer W and kept on ice. MBP-ASC (350 ng) was added to 20 μl of beads and incubated on ice for 1 h. Amylose beads alone or bound to MBP or MBP-ASC were added 1:1 with ASC KO lysate and incubated at 37 °C for 30 min.

### MBP-ASC expression and purification

The full size human ASC 22 kDa complementary DNA variant was PCR amplified and inserted into pMAL-C2X vector plasmid (New England Biolabs) creating a chimeric protein linking MBP to the PYD of ASC. After sequence validation, the plasmid was transformed to *E. coli* Rosetta to produce the MBP-ASC fusion protein. Bacteria cultures were grown overnight in LB media containing 50 μg/ml ampicillin, 50 μg/ml chloramphenicol, and 1% glucose. When the bacterial culture reached density of 2 × 10^8^/ml (absorbance at 600 nm = 0.5), IPTG was added (0.5 mM final concentration) and incubated for another 3 h. Then bacteria were pelleted by centrifugation at 4000*g* for 15 min, and pellet was resuspended in a harvest buffer (20 mM Tris–HCl, pH 7.4, 200 mM NaCl, 1 mM EDTA, and 1 mM DTT supplemented with a protease inhibitor cocktail, PMSF) and neutrophil elastase inhibitor methoxy-succinyl-ala-ala-pro-val-chloromethyl ketone. The bacterial pellet was sonicated on ice and then centrifuged at 10,000*g* for 10 min at 4 °C. The resulting supernatant was loaded onto an amylose-bead resin column (New England Biolabs) at a flow rate of 0.5 ml/min. After the column was washed with 10 volumes of the harvest buffer deplete of the protease inhibitor supplements, MBP-ASC was then eluted from the amylose beads with harvest buffer containing 10 mM maltose. The eluted protein concentration was measured by UV absorbance at 280 nm using a Nanodrop 2000 (Thermo Fisher Scientific). MBP-ASC was verified by immunoblot with both anti-MBP antibody (New England Biolabs) and rabbit antiASC polyclonal antibody (generated in our laboratory). Eluted proteins were stored at −80 °C.

### ASC crosslinking and Western blots

Samples were crosslinked, and immunoblots were performed as previously described (21). Briefly, samples were diluted 1:10 with prechilled PBS and then split. Half of the sample was immediately prepared for immunoblot by addition of Laemmli buffer containing 10% 2-mercaptoethanol (Bio-Rad) and referred as “total ASC,” and the second half was incubated at room temperature with 2 mM disuccinimidyl suberate (Thermo Fisher Scientific) for 30 min before the reaction was quenched with Laemmli buffer, referred to as “cross-linked ASC.” Samples were run on a 4 to 12% Bis–Tris gel (Invitrogen), and proteins were separated using SDS-PAGE, then transferred to a polyvinylidene difluoride membrane, pore size 0.45 μm (Merck Millipore). After blocking in 5% milk in Tris-buffered saline (Thermo Fisher Scientific) containing 1% Tween-20 (Sigma) (TBST), polyvinylidene difluoride membranes were washed for 5 min three times in TBST, and then probed with either ASC, IL-1β, or caspase-1 antibodies, all developed in our laboratory with their specificity confirmed using respective recombinant proteins as previously described (35). Membranes were then washed extensively in TBST wash and probed with either sheep antimouse or donkey anti-rabbit antibody tagged with horseradish peroxidase (GE Healthcare). Blots were developed with enhanced chemiluminescence reagent (Thermo Fisher Scientific). Reblotting was done with Re-Blot Plus Mild Solution (MilliporeSigma) followed by TBST wash and subsequent blocking, primary antibody, secondary antibody, and enhanced chemiluminescence development.

### IL-1β ELISA, LDH assay, and caspase-1 activity assay

IL-1β ELISA and LDH quantifications were performed as previously described (35). Briefly, THP-1, ASC KO, WT-ASC KI, and Y146A-ASC KI were incubated at 106 cells/ml in RPMI1640 supplemented with 10% fetal bovine serum and 1% penicillin/streptomycin with 1 μg/ml LPS (InvivoGen) for 3 to 4 h and ATP (Sigma–Aldrich) at 5 mM for the last 30 min. Cells were then pelleted and separated from media by 500*g* spin for 5 min. Media were collected and assayed for cell death by measuring LDH and IL-1β release. As we described earlier (36), after incubation with LPS and ATP, cells were pelleted at 600*g* for 5 min, and cell supernatant was used to determine percent of LDH release as indicator of cell death. To determine spontaneous cell death, we collected cell medium incubated for the same time in the absence of LPS + ATP stimulation. Total LDH concentration in cells was referred as positive control and was measured in cells lysed with Triton X-100 (1% final concentration). Cell culture alone was used as a blank, and absorbance values were subtracted from reading of the unknown samples and positive control (% LDH release = [sample/positive control] × 100). LDH concentration was detected at a wavelength of 490 nm. IL-1β release was detected by sandwich ELISA (clone 2805 as a coating antibody; R&D Systems, and a rabbit polyclonal anti-IL-1β antibody developed in our laboratory as detection antibody). To quantity kinetic activity of caspase-1, we measured total protein in the cell extract of each cell type *via* DC Lowry Assay (Bio-Rad). Following the caspase-1 activation protocol (Fig. 1*H*), we assumed equal protein concentration between the 37 °P and the 4 °S fractions and equalized protein concentration to 30 μg/50 μl of lysis buffer W, which was then added to 50 μl lysis buffer W + 100 μM Ac-WEHD-afc in a 96-well black Costar plate (Corning). Caspase cleavage of WEHD-afc was measured at room temperature *via* CytoFluor Series 4000 Fluorescence Multi-Well Plate Reader (excitation of 360/40 nm, emission of 460/40 nm) with 30 readings over 30 min.

### Statistical analysis

All experiments were performed independently three or more times (as indicated in the legends to the figures) and ELISA, LDH, and WEHD-afc cleavage results expressed as mean values ± SEM, as indicated in the legends to the figures. Comparison of groups for statistical difference was done using Student’s *t* test and *p* value <0.05 was considered to be significant.

## Data availability

All data generated for this study are included within this article.

## Supporting information

This article contains supporting information.

## Conflict of interest

The authors declare that they have no conflicts of interest with the contents of this article.
