# Supplementary material for: The central inflammasome adaptor protein ASC activates the inflammasome after transition from a soluble to an insoluble state
Source: J Biol Chem. 2022 May 11;298(6):102024. doi: 10.1016/j.jbc.2022.102024 (PMC9163591; doi:10.1016/j.jbc.2022.102024)
Supplement: Supporting Information [file mmc1.pdf]

The central inflammasome adaptor protein ASC activates the inflammasome after transition from a soluble to an insoluble state

Evan R. Prather[1], Mikhail A. Gavrilin[1,2] and Mark D. Wewers[1,2]

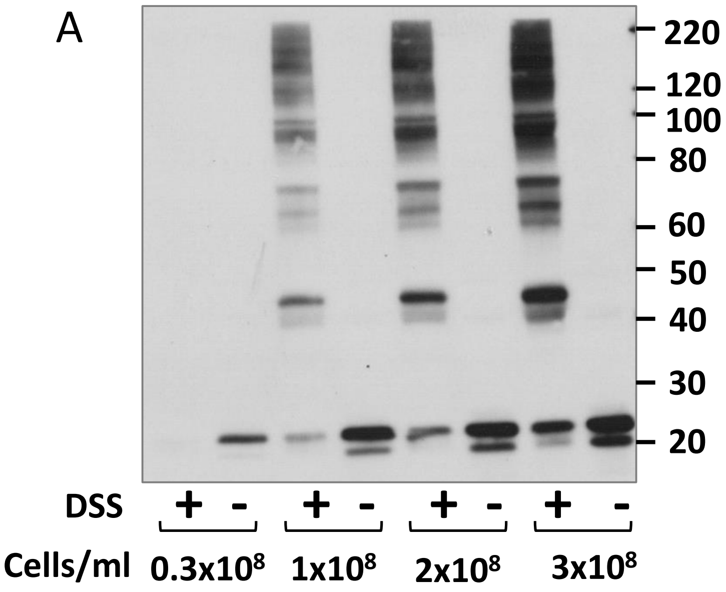

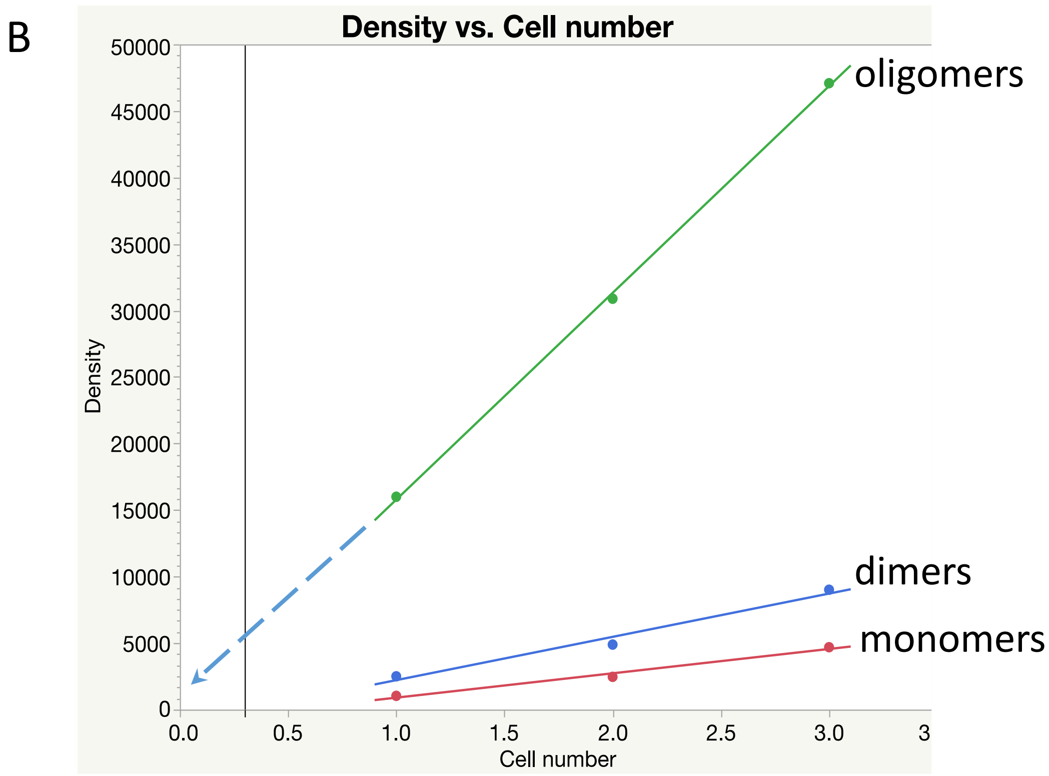

C

ASC Form	Linear Fit: y=mx+b	R^2	Predicted Density For 0.3 x 10* cells/ml
monomer	1828x - 930	0.98	undetectable
dimer	3253x - 1038	0.98	undetectable
oligomer	15543x + 235	1.00	4897

Supporting Information. ASC oligomer formation dependence upon cell lysing concentration. ASC Immunoblot. THP cells were syringe-lysed at differing concentrations (0.3 – 3.0x10^8 cells/ml) in lysis buffer as outlined in Figure 1C. Cell extracts were then stored at 4°C or warmed at 37°C for 30 min before 2 mM disuccinimidyl suberate (DSS) crosslinking and then immunoblotted. Immunoblots were probed with anti-ASC (rabbit polyclonal antiserum) (**A**). Densitometric measures of DSS crosslinked ASC were subjected to linear regression analysis for concentrations ≥ 1.0x10^8 cells/ml (**B**). Predictions support Immunoblot sensitivity to detect DSS ASC oligomers if present at 0.3x10^8 cells/ml (**C**).
